# Intragastric balloon for obesity treatment: Systematic review and meta-analysis of randomized controlled trials

**DOI:** 10.1055/a-2681-2859

**Published:** 2025-09-03

**Authors:** Gabriel de Oliveira Amaral, João Pedro Schmitt, Lucas Monteiro Delgado, Gilmara Coelho Meine

**Affiliations:** 1 125099 Department of Medicine, FEEVALE University, Novo Hamburgo, Brazil; 2 28114 Medicine, Universidade Federal de Minas Gerais, Belo Horizonte, Brazil; 3 125098 Health Sciences Institute / Medical School, Universidade FEEVALE, Novo Hamburgo, Brazil

**Keywords:** Endoscopy Upper GI Tract, Statistics, Epidemiology

## Abstract

**Background and study aims:**

Intragastric balloon (IGB) is a minimally invasive and reversible endoscopic option for treating obesity. This systematic review and meta-analysis compared the effectiveness of IGB versus standard medical therapy (SMT) for obesity management, including studies with a minimum treatment duration of 6 months. Subgroup analyses were performed based on IGB type, baseline mean body mass index (BMI), and use of pharmacological therapy in the SMT group.

**Methods:**

We searched for randomized controlled trials (RCTs) in MEDLINE, EMBASE, and Cochrane Library databases. Outcomes were evaluated at 6, 9, and 12 months after initiation of treatment. A random-effects model was used to calculate the pooled mean difference (MD) with 95% confidence interval (CI) for continuous outcomes.

**Results:**

We included 15 RCTs (1961 patients). Compared with SMT, IGB significantly improved the percentage of excess weight loss at 6 months (MD 16.80; 95% CI 9.22–24.38), 9 months (MD 14.36; 95% CI 7.67–21.04), and 12 months (MD 13.10; 95% CI 10.43–15.77). IGB also showed superior results in percentage of total weight loss, absolute weight loss, and BMI reduction at all time points compared with SMT. There were significant subgroup differences for some outcomes according to IGB type and baseline mean BMI.

**Conclusions:**

In obese adults, IGB is more effective than SMT for weight loss at 6, 9, and 12 months.

## Introduction

Nearly half of US adults are projected to be affected by obesity by 2030 1 . Obesity-related conditions, such as cardiovascular disease, type 2 diabetes mellitus, chronic joint disease, and obstructive sleep apnea, significantly contribute to morbidity and mortality 2 . The primary approach to weight loss involves standard medical therapy (SMT), which includes dietary modifications, physical activity, and pharmacological therapies. Although more invasive, bariatric surgery is a highly effective option for achieving sustained weight loss and long-term remission of obesity-related comorbidities 3 . In recent years, endoscopic sleeve gastroplasty has emerged as a minimally invasive alternative, demonstrating efficacy and a favorable safety profile, despite limited data from randomized controlled trials (RCTs) 4 .

**Fig. 1:**
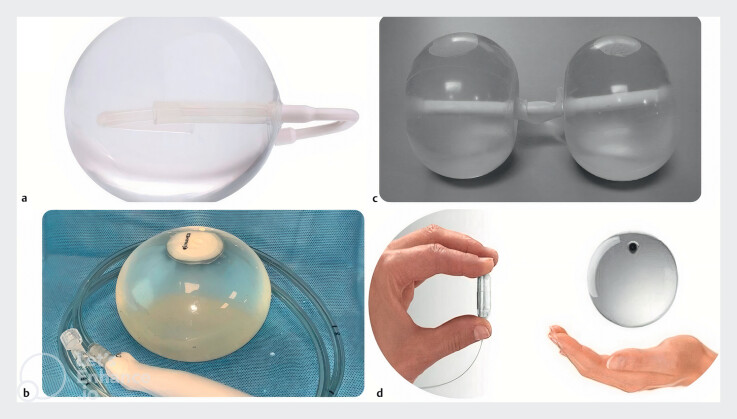
Types of intragastric balloons included in the meta-analysis. **a** Spatz3 Balloon (adapted from Stavrou et al. World J Gastrointest Endosc 2021; 13: 238–259). **b** ReShape Duo Balloon (adapted from Ponce et al. Surg Obes Relat Dis 2015; 11: 874–881). **c** Orbera Balloon (adapted from Stavrou et al. World J Gastrointest Endosc 2021; 13: 238–259). **d** Obalon Balloon (adapted from Sullivan et al. Surg Obes Relat Dis 2018; 14: 1876–1889).

However, some patients do not respond to SMT and do not meet the criteria for bariatric surgery or choose not to undergo it due to its invasive and irreversible nature 5 6 . In this context, intragastric balloons (IGBs) are another minimally invasive and reversible option for treating obesity 7 . There are several types of IGBs, which differ by design, filling material, method of placement, and approved duration of use ( Fig. 1 ). Although uncertainty remains regarding sustained weight loss, the procedure represents a promising therapeutic alternative for managing obesity 8 9 10 .

Three previous meta-analyses of RCTs have assessed efficacy of IGB at the end of the treatment. Moura et al. 11 reported no significant difference in percentage of excess weight loss (%EWL) between IGB and sham/diet in their quantitative analysis. In contrast, Kotinda et al. 12 and Saber et al. 9 found significantly greater reductions in %EWL with IGB compared with lifestyle intervention (LI). The discrepancy between these findings and the limited long-term data highlights the need for further investigation.

Thus, we conducted a systematic review and meta-analysis of RCTs evaluating efficacy and safety of IGB compared with SMT for obesity treatment, with outcomes assessed at 6, 9, and 12 months. We aimed to evaluate IGB therapy with a duration of at least 6 months. In addition, we performed subgroup analyses based on type of balloon used, baseline mean body mass index (BMI), and use of pharmacological therapy in the SMT group.

## Methods

### Protocol and registration

We conducted this systematic review and meta-analysis in accordance with the Cochrane Handbook for Systematic Reviews of Interventions and structured it according to the Preferred Reporting Items for Systematic Reviews and Meta-Analysis (PRISMA) recommendations 13 14 ( **Supplementary Table 1** ). This study was registered in the International Prospective Register of Systematic Reviews (PROSPERO) under protocol CRD42024584532.

### Study outcomes and additional analyses

Primary and secondary outcomes were prespecified in the study protocol, based on their clinical relevance and the frequency with which they were reported in the identified studies during the initial viability assessment, which may not necessarily reflect the original hierarchy of outcomes in the primary studies.

The primary outcome was %EWL. Secondary outcomes were: 1) percentage of total body weight loss (%TBWL); 2) absolute weight loss (AWL); and 3) BMI reduction. Outcomes were assessed at 6, 9, and 12 months.

Aiming to evaluate potential sources of heterogeneity, we conducted prespecified subgroup analyses based on the diverse types of IGB (Orbera, Obalon, ReshapeDuo, and Spatz3), mean baseline BMI (≤ 40 mg/m ^2^ and > 40 kg/m ^2^ ), and use of pharmacologic therapy in the control group (LI with pharmacologic therapy and LI without pharmacologic therapy). Moreover, we performed a meta-regression analysis using mean baseline BMI as a covariate.

### Eligibility criteria

We selected studies based on the following inclusion criteria: 1) RCTs; 2) comparing IGB with SMT; 3) with treatment duration of at least 6 months; and 4) reporting at least one of the outcomes of interest. No restrictions were applied regarding type of IGB or publication language. Only studies published as full-text peer-reviewed articles were included. Editorials, abstracts, letters, reviews, systematic reviews, and meta-analyses were excluded.

### Search strategy and data extraction

We searched the PubMed, Embase, and Cochrane Library databases to identify studies that met the inclusion criteria and were published up to September 2024. The search strategy is detailed in **Supplementary Table 2** . Two reviewers (J.P.S. and G.O.A.) conducted the search, imported results into Zotero 6.0, and triaged the studies. After exclusion of duplicates and articles with titles/abstracts clearly not related to the clinical question, eligibility of each remaining study was assessed based on the review of the full-text articles. We also searched for additional studies in the references of the included RCTs, as well as in previous systematic reviews and meta-analyses. Disagreements were solved by a third author (G.C.M.). Interrater reliability during the study selection process was assessed using Cohen’s Kappa coefficient, calculated between two independent reviewers.

Two authors (G.O.A. and J.P.S.) extracted the data into a standardized format, including study characteristics (first author, year of publication, country, study design, and sample size); participant characteristics (age, sex, BMI, weight); procedure characteristics (type of IGB and time of balloon duration); and SMT characteristics (LI with or without pharmacological therapy).

### Risk of bias and evidence quality assessment

Based on the Cochrane Risk of Bias 2 (RoB-2) tool 15 , two independent examiners (J.P.S. and G.O.A.) evaluated the selected studies and derived overall risk of bias assessment. We assessed publication bias using funnel plots only for outcomes with at least 10 studies because the power of this test is insufficient to discriminate between chance and true funnel plot asymmetry when analyzing fewer studies 16 . We assessed certainty of the evidence for each outcome using the Grading of Recommendations, Assessment, Development, and Evaluation (GRADE) tool 17 . For each outcome, we rated certainty of the evidence as high, moderate, low, or very low.

### Data analysis

We used the DerSimonian and Laird random-effects model to calculate the pooled mean difference of continuous outcomes with 95% confidence intervals (CIs) and prediction intervals. *P* < 0.05 was considered statistically significant. If studies did not provide the mean and standard deviation of the sample, data were calculated based on the sample’s reported median and range, according to the methods devised by Luo et al. and Wan et al. 18 19 . The extent of heterogeneity among the observed effect estimates was quantified by between-study variance, as represented by Tau ^2^ . We conducted a leave-one-out sensitivity analysis in cases of heterogeneity. In the subgroup analysis, *P* < 0.10 was considered indicative of significant treatment interaction. Effect estimates and heterogeneity in pair-wise meta-analysis were calculated using Review Manager 5.4 (Nordic Cochrane Centre, The Cochrane Collaboration, Copenhagen, Denmark). We calculated pooled means with 95% CIs for each group, prediction interval for pair-wise meta-analysis, and conducted meta-regression analysis using R software (version 4.2.1; R Foundation for Statistical Computing).

## Results

### Study selection and characteristics

**Fig. 2:**
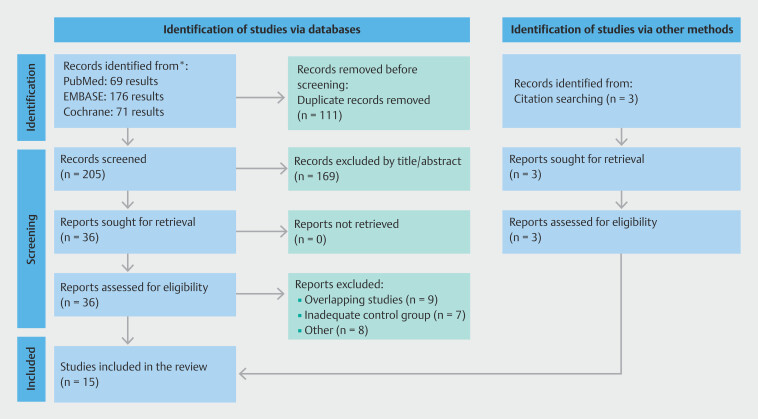
PRISMA flow diagram of study screening and selection 14 .

As detailed in Fig. 2 , the initial search yielded 316 results. After removing duplicates and ineligible studies, 36 remained and were fully reviewed based on the inclusion criteria. Ultimately, 12 RCTs 20 21 22 23 24 25 26 27 28 29 30 31 were included, along with three additional studies 32 33 34 identified through citation searching. Cohen’s Kappa coefficient during the study selection process was 0.883 (standard error = 0.019; 95% CI 0.846- 0.919), indicating substantial agreement. The 15 RCTs comprised a total of 1961 patients from 10 countries, with 1078 (55%) assigned to IGB therapy and 883 (45%) to SMT. All studies included LI as part of the SMT regimen, whereas two studies 20 32 also used pharmacological therapy with sibutramine. In addition, five studies 24 25 29 31 32 included a sham procedure as part of the SMT group. In the IGB group, treatment duration was 6 months in 14 trials 20 21 22 23 24 25 26 27 28 29 31 32 33 34 and 8 months in one trial 30 . A total of 12 studies 20 21 23 24 26 27 28 29 30 31 32 33 used fluid-filled balloons, one 25 used air-filled balloons, one 34 used fluid-filled or air-filled balloons, and one did not specify 22 . Only two studies 22 34 did not describe LI as part of treatment in the intervention group. Detailed baseline characteristics of the included studies are described in Table 1 .

**Table:** **Table 1** Baseline characteristics of included studies.

**First author, year**	**Design**	**Country**	**Intervention**	**SMT**	**Treatment duration, months**	**Follow-up, months**	**Sample size IGB | SMT**	**Age, years (SD)**	**Female,% IGB | SMT**	** Baseline BMI, kg/m ^2^ (SD) IGB | SMT **
Abu Dayeh, 2021	RCT	USA	IGB Spatz 3 fluid-filled + LI	LI	8	14	187 | 101	44.4 (8.9) | 44.0 (8.9)	87 | 89	35.8 (2.6) | 35.8 (2.7)
Fuller, 2013	RCT	Australia	IGB Orbera fluid-filled + LI	LI	6	12	37 | 37	43.4 (9.4) | 48.1 (7.3)	68 | 66	36.0 (2.7) |36.7 (2.9)
Kashani, 2022	RCT	Iran	IGB	LI	6	6	34 | 34	36.6 (11.2) | 33.9 (8.8)	85 | 82	38.9 (6.6) | 43.2 (7.0)
Gómez, 2016	RCT	USA	IGB Orbera fluid-filled + LI	LI	6	13	15 | 14	38.1 (8.8) | 38.2 (8.78)	87 | 93	34.7 (3.4) | 35.6 (2.8)
Mohammed, 2014	RCT	Egypt	IGB Orbera fluid-filled	LI	6	9	84 | 44	43.9 (8.9) | 42.6 (6.6)	43 | 41	47.8 (1.0) | 47.4 (1.0)
Courcoulas, 2017	RCT	USA	IGB Orbera fluid-filled + LI	LI	6	12	125 | 130	38.7 (9.3) | 40.8 (9.6)	90 | 90	35.0 | 35.0
Farina, 2012	RCT	Italy	IGB Orbera fluid-filled + LI	LI + sibutramine	6	12	30 | 20	36.6 (1.5) | 32.7 (1.8)	77 | 80	42.3 (1.0) | 41 (1.3)
Lee, 2012	RCT	Singapore	IGB Orbera fluid-filled + LI	LI+ sham	6	6	8 | 10	43.0 (19.7)* | 47.0 (15.0)*	62 | 20	30.3 (5.7) | 32.4 (9.1)
Hollenbach, 2024	RCT	Germany	IGB Orbera fluid-filled + LI	Sham + LI	6	24	15 | 7	42.2 (12.3) | 38.7 (15.0)	47 | 57	39.3 (4.2) | 44.1 (6.2)
Sullivan, 2018	RCT	USA	IGB Obalon air-filled + LI	LI + Sham	6	6	198 | 189	42.7 (9.6) | 42.5 (9.3)	86 | 90	35.2 (2.7) | 35.5 (2.7)
Ponce, 2015	RCT	USA	IGB ReShape Duo fluid-filled + LI	LI + Sham	6	6	187 | 139	43.8 (9.5) | 44.0 (10.2)	95 | 95	35.3 (2.8) | 35.4 (2.6)
Vicente, 2020	RCT	Spain	IGB Orbera fluid-filled + LI	LI	6	6	32 | 34	43 (10.2) | 42.6 (9.2)	66/71	46.4 (10.4) | 46.0 (8.5)
Chan, 2021	RCT	Australia	IGB Orbera fluid-filled + LI	LI + sham + sibutramine	6	10†	50 | 49	38.1 (7.9) | 35.3 (7.2)	70/75	30.2 (2.3) | 30.7 (2.1)
Coffin, 2017	RCT	France	IGB Orbera fluid-filled or air-filled	LI	6	12	55 | 60	40.5 (12.3) | 40.1 (11.6)	76/72	53.9 (6.5) | 54.7 (10.3)
Konopko-Zubrzycka, 2009	RCT	Poland	IGB Orbera fluid-filled + LI	LI	6	10	21 | 15	41 (11.9) | 42.8 (9.4)	48/60	47.3 (5.7) | 47.1 (6.9)
*Median (interquartile range). †Years. BMI, body mass index; IGB, intragastric balloon; LI, lifestyle intervention; RCT, randomized controlled trial; SD, standard deviation; SMT, standard medical treatment										

### Primary outcome

**Fig. 3:**
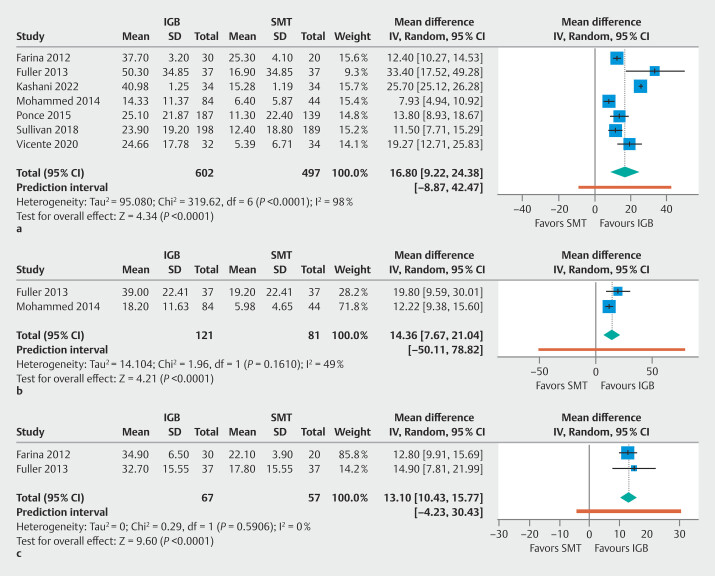
Forest plots of percentage of excess weight loss at **a** 6 months, **b** 9 months, and **c** 12 months. The IGB group had a significantly higher %EWL compared with the SMT group. %EWL, percentage of excess weight loss; CI, confidence interval; IGB, intragastric balloon; SMT, standard medical treatment.

Weight loss in each group and pooled effect estimates are summarized in Table 2 . Compared with the SMT, the IGB group had a significantly higher %EWL at 6 months (7 studies 20 21 22 23 24 25 26 ; n = 1099; MD 16.80%; 95% CI 9.22–24.38; *P* < 0.0001; Tau² = 95.08; Table 2 ; Fig. 3 **a** ), 9 months (2 studies 21 23 ; n = 202; MD 14.36%; 95% CI 7.67–21.04; *P* < 0.0001; Tau² = 14.10; Table 2 ; Fig. 3 **b** ), and 12 months (2 studies 20 21 ; n = 124; MD 13.10%; 95% CI 10.43–15.77; *P* < 0.0001; Tau² = 0.00; Table 2 ; Fig. 3 **c** ).

**Table:** **Table 2** Results from pooled analyses for primary and secondary outcomes.

**Outcome**	**Number of studies (patients)**	**Single-arm analysis, mean (95% CI)**		**Two-arm analysis, effect estimate (95% CI)**	***P* value **
		**IGB group**	**SMT group**		
%EWL 6 months	7 (1099)	30.37 (23.25–37.48)	13.07 (8.17–17.98)	MD 16.80 (9.22–24.38)	< 0.001
%EWL 9 months	2 (202)	28.31 (7.93–48.68)	12.09 (-0.82–25.01)	MD 14.36 (7.67–21.04)	< 0.001
%EWL 12 months	2 (124)	34.51 (32.40–36.62)	20.62 (16.61–24.62)	MD 13.10 (10.43–15.77)	< 0.001
%TBWL 6 months	8 (1208)	11.01 (7.95–14.08)	4.70 (2.58–6.81)	MD 5.82 (4.42–7.23)	< 0.001
%TBWL 9 months	3 (617)	13.11 (9.80–16.41)	3.78 (2.72–4.83)	MD 7.66 (3.34–11.98)	< 0.001
%TBWL 12 months	5 (430)	9.70 (6.13–13.27)	4.63 (1.84–7.42)	MD 5.33 (4.16–6.50)	< 0.001
AWL 6 months, kg	8 (1126)	12.35 (9.83–14.87)	5.06 (3.67–6.45)	MD 6.98 (4.80–9.16)	< 0.001
AWL 9 months, kg	4 (745)	13.14 (8.82–17.45)	4.37 (2.91–5.83)	MD 8.65 (4.93–12.37)	< 0.001
AWL 12 months, kg	4 (478)	10.175 (4.61–15.73)	5.38 (1.97–8.78)	MD 4.87 (1.87–7.88)	0.001
BMI loss 6 months, kg/m²	8 (1182)	3.51 (2.93–4.23)	1.17 (0.75–1.60)	MD 2.27 (1.53–3.01)	< 0.001
BMI loss 12 months, kg/m²	2 (124)	4.85 (2.01–7.69)	2.61 (1.24–3.98)	MD 2.27 (0.80–3.74)	< 0.001
AWL, absolute weight loss; BMI, body mass index; CI, confidence interval; IGB, intragastric balloon; MD, mean difference; SMT, standard medical treatment; %EWL, percentage of excess weight loss; %TBWL, percentage of total body weight loss.					

### Secondary outcomes

#### TBWL

The IGB group had a significantly higher %TBWL at 6 months (8 studies 20 21 24 25 26 27 28 29 ; n = 1208; MD 5.82%; 95% CI 4.42–7.23; *P* < 0.0001; Tau² = 2.62; Table 2 ; **Supplementary Fig. 1a** ), 9 months (3 studies 21 27 30 ; n = 617; MD 7.66%; 95% CI 3.34–11.98; *P* = 0.0005; Tau² = 13.44; Table 2 ; **Supplementary Fig. 1b** ), and 12 months (5 studies 20 21 27 28 29 ; n = 430; MD 5.33%; 95% CI 4.16–6.50; *P* < 0.0001; Tau² = 0.48; Table 2 ; **Supplementary Fig. 1c** ) compared with SMT.

#### AWL

Similarly, the AWL was significantly higher in patients treated with IGB in comparison with SMT at 6 months (8 studies 21 22 23 25 26 27 32 33 ; n = 1126; MD 6.98 kg; 95% CI 4.80–9.16; *P* < 0.0001; Tau² = 7.68; Table 2 ; **Supplementary Fig. 2a** ), 9 months (4 studies 21 23 27 30 ; n = 745; MD 8.65 kg; 95% CI 4.93–12.37; *P* < 0.0001; Tau² = 13.46; Table 2 ; **Supplementary Fig. 2b** ), and 12 months (4 studies 20 21 27 32 ; n = 478; MD 4.87 kg; 95% CI 1.87–7.88; *P* = 0.001; Tau² = 8.28; Table 2 ; **Supplementary Fig. 2c** ).

#### BMI loss

BMI loss was reported at 6 and 12 months. Compared with SMT, IGB significantly improved this outcome at 6 months (8 studies 21 22 23 24 25 26 31 34 ; n = 1182; MD 2.27 kg/m²; 95% CI 1.53–3.01; *P* < 0.0001; Tau² = 0.79; Table 2 ; **Supplementary Fig. 3a** ) and 12 months (2 studies 20 21 ; n = 124; MD 2.27 kg/m²; 95% CI 0.80–3.74; *P* = 0.0007; Tau² = 1.03; Table 2 ; **Supplementary Fig. 3b** ).

### Leave-one-out sensitivity analysis

We conducted leave-one-out sensitivity analyses for outcomes that demonstrated between-study variance ( **Supplementary Table 3** ). In the analysis, no single study was found to excessively influence the effect estimate or drive heterogeneity across the majority of the evaluated outcomes. The only exceptions were %TBWL at 9 months and 12 months. For the outcome %TBWL at 9 months, excluding the study by Abu Dayyeh et al. 30 , which was the only trial with an 8-month IGB treatment duration, eliminated the heterogeneity. For the outcome %TBWL at 12 months, heterogeneity was eliminated by excluding the study by Farina et al. 20 , which was the only trial in this outcome that included pharmacological therapy with sibutramine. In both cases, their exclusion did not significantly impact the effect estimate.

### Subgroup analysis

#### Type of IGB

Subgroup analyses based on the type of IGB (Orbera, Spatz 3, ReShapeDuo, and Oballon) are described in **Supplementary Fig. 4** , **Supplementary Fig. 5** , **Supplementary Fig. 6** , **Supplementary Fig. 7** , and **Supplementary Fib. 8** . There were significant subgroup differences in %TBWL at 6 months ( *P* = 0.0003; **Supplementary Fig. 5** ), %TBWL at 9 months ( *P <* 0.00001; **Supplementary Fig. 6** ), and AWL at 6 months ( *P* < 0.01; **Supplementary Fig. 7** ).

#### Baseline BMI

The subgroup analysis by baseline BMI (≤ 40 kg/m ^2^ and > 40 kg/m ^2^ ) is described in **Supplementary Fig. 9** , **Supplementary Fig. 10** , **Supplementary Fig. 11** , and **Supplementary Fig. 12** . There were significant subgroup differences for the outcomes %TBWL at 6 months ( *P* = 0.04; **Supplementary Fig. 10** ) and AWL at 6 months ( *P* = 0.0005; **Supplementary Fig. 11** ). In the subgroup of studies in which patients had a mean baseline BMI > 40, there was a significantly higher %TBWL and AWL at 6 months.

#### Use of pharmacologic therapy in the control group

The subgroup analysis based on use of pharmacologic therapy in the control group (LI with pharmacologic therapy and LI without pharmacologic therapy) was possible only for the outcome of AWL at 12 months, and there was no significant treatment interaction ( *P* = 0.77; **Supplementary Fig. 13** ).

### Meta-regression analysis

Meta-regression analysis using mean baseline BMI as a covariate showed that there was no significant relationship between mean baseline BMI and relative efficacy of IGB compared with SMT for the outcomes of %EWL and %TBWL within 6 months and %TBWL within 12 months ( **Supplementary Fig. 14** , **Supplementary Fig. 15** , and **Supplementary Fig. 16** ). However, patients with higher mean baseline BMI were more likely to experience greater AWL and BMI reduction within 6 months ( **Supplementary Fig. 17** and **Supplementary Fig. 18** ).

### Risk of bias and evidence quality assessment

Results of bias risk assessment according to the RoB-2 tool are presented in **Supplementary Table 4** . A total of five studies were considered to have some concerns regarding the randomization process, deviations from the intended interventions, and selection of the reported results. We did not assess publication bias because of the limited number of studies in each outcome. The summary of findings and GRADE assessment is shown in **Supplementary Table 5** .

## Discussion

This systematic review and meta-analysis included 15 studies with a total of 1961 patients, comparing IGB therapy with SMT for obesity management. The IGB group showed significant improvements in %EWL, %TBWL, AWL, and BMI reduction at all follow-up intervals (6, 9, and 12 months).

In the present study, all but one trial reported IGB removal in 6 months. The exception was the study by AbuDayeh et al. 30 , in which the IGB remained in place for 8 months; however, this study did not report weight loss outcomes at 12 months. Therefore, the 12-month results reflect weight changes observed 6 months after balloon removal.

IGB therapy is a reversible weight-loss method, and beyond its well-established effect of reducing gastric space 10 , studies suggest that it may also reduce secretion of appetite-regulating hormones, alter carbohydrate and lipid metabolism, and modify gastric motility. These additional mechanisms further enhance weight-loss effects of IGB 23 28 .

Previous meta-analyses of RCTs have compared IGB therapy with SMT for treating overweight and obese patients 9 11 12 . Moura et al. 11 reported no significant difference in %EWL between the groups in quantitative analysis, whereas Kotinda et al. 12 found significantly greater %EWL reductions at the end of treatment with IGB. Both studies had a smaller number of RCTs compared with the present study and included trials in which IGBs were used for less than 6 months 11 12 .

Another meta-analysis by Saber et al. 9 included 20 RCTs with a total of 1195 patients, reporting significant improvements in the IGB group for BMI loss, %EWL, AWL, and %TBWL at 3 months. However, many of the included studies utilized IGBs that are no longer used in current clinical practice.

In contrast to previous meta-analyses, our study included more recent trials with longer IGB treatment (≥ 6 months), which may better reflect standard clinical use of IGB today. In addition, we had a larger sample size, stratified results at 6, 9, and 12 months since treatment initiation, and conducted subgroup analyses to explore potential sources of heterogeneity.

International societies (American Gastroenterological Association, American Society for Gastrointestinal Endoscopy, and European Society of Gastrointestinal Endoscopy) suggest using IGB therapy associated with LI over LI alone in obese individuals who have failed a trial of conventional weight-loss strategies 35 36 .

IGBs yielded superior results in all assessed outcomes compared with SMT, but our subgroup analysis provided important insights. First, there were significant subgroup differences among the diverse IGB types. Second, the subgroup of patients with mean baseline BMI > 40 kg/m ^2^ exhibited a significantly higher %TBWL at 6 months than patients with mean baseline BMI ≤ 40 kg/m ^2^ . In addition, for the outcome of AWL at 12 months, subgroup analysis based on use or non-use of pharmacologic therapy with sibutramine in the control group showed no significant difference between the subgroups.

After IGB removal, AGA suggests subsequent or maintenance interventions for weight loss, which include dietary interventions, pharmacotherapy, repeat IGB, or bariatric surgery. The method of choice is determined based on the patient's context and comorbidities, following a multidisciplinary, shared decision-making approach that includes nutritionists and mental health professionals 35 . Furthermore, it is essential to monitor the patient’s progress and adjust the interventions as necessary to ensure that IGB treatment results are maintained for as long as possible.

One of the included RCTs randomly assigned patients in the IGB group to receive either LI plus sibutramine 10 mg or LI alone for an additional 6 months after IGB removal. At 12 months (6 months post-IGB removal), there was no statistically significant difference in overall weight loss between LI plus sibutramine and LI. However, a trend toward greater %TBWL was observed among those receiving maintenance with LI plus sibutramine after IGB removal. This study underscores the importance of intensive LI, either alone or in combination with pharmacological therapy, for maintaining weight loss after IGB removal.

New pharmacotherapies, such as glucagon-like peptide-1 receptor agonists (GLP-1RAs), have recently emerged for treatment of both type 2 diabetes and obesity. Despite their high cost and the need for ongoing use, GLP-1RAs have gained popularity due to their long-term effectiveness 37 . Two recent retrospective studies evaluated liraglutide: one compared it with IGB therapy 38 , whereas the other assessed its impact when combined with IGB 39 .

Martines et al. 38 compared IGB insertion vs liraglutide prior to laparoscopic sleeve gastrectomy (LSG). The IGB group had a higher %EWL at 6 months and 12 months compared with the liraglutide group. Ultimately, the findings of this study demonstrate that, despite IGB and Liraglutide yielding significant results in preoperative treatment prior to LSG, use of IGB consistently outperformed pharmacotherapy in terms of both early and sustained weight loss. The authors suggest that IGBs should be considered as a viable alternative regarding preoperative management of super-obese patients.

Yilmaz et al. 39 compared IGB vs IGB plus liraglutide. The group that received combined therapy had higher AWL and BMI reduction at 6 months compared with those who received IGB alone. However, when the outcomes were stratified by gender, no significant difference was observed between the groups.

Our study has some limitations. First, significant heterogeneity was observed in most outcomes, which persisted in some cases despite subgroup and sensitivity analyses. Second, studies employed diverse measurements for assessing weight loss, and we included multiple metrics based on the data provided in the literature. The large number of outcomes and multiple time points evaluated, as well as the various subgroup analyses, introduce a potential risk of multiplicity-related bias. Although efforts were made to mitigate this issue by prespecifying clinically relevant analyses and conducting meta-regression and sensitivity analyses, multiplicity-related bias cannot be entirely excluded. Third, although our primary outcome was %EWL, we acknowledged that %TBWL and BMI reduction demonstrated lower heterogeneity (as demonstrated by lower Tau² values) compared with %EWL and AWL at most time points, which may support their use as more consistent and standardized measures in future research. Finally, due to limited data availability, we were unable to evaluate cardiometabolic parameters.

## Conclusions

In conclusion, this systematic review and meta-analysis of RCTs demonstrated that IGB therapy is more effective than SMT alone for treating obesity. By significantly improving all weight-loss metrics at 6, 9, and 12 months, IGB is a valuable tool for weight management, especially for individuals who have not achieved satisfactory results with SMT alone, offering a minimally invasive treatment alternative.

## Supplementary Material

Supplementary Material Zusatzmaterial
