# Supplementary material for: Intragastric balloon for obesity treatment: Systematic review and meta-analysis of randomized controlled trials
Source: Endosc Int Open. 2025 Sep 3;13:a26812859. doi: 10.1055/a-2681-2859 (PMC12417771; doi:10.1055/a-2681-2859)
Supplement: Supplementary file 1 — Supplementary Material [file 10-1055-a-2681-2859_26846707.pdf]

Supplementary Table 1 PRISMA Statement Checklist [14].

Topic	No.	Item	Location where item is reported
**TITLE**			
**Title**	1	Identify the report as a systematic review.	Page 1
**ABSTRACT**			
**Abstract**	2	See the PRISMA 2020 for Abstracts checklist	
**INTRODUCTION**			
**Rationale**	3	Describe the rationale for the review in the context of existing knowledge.	Page 2
**Objectives**	4	Provide an explicit statement of the objective(s) or question(s) the review addresses.	Page 2-3
**METHODS**			
**Eligibility criteria**	5	Specify the inclusion and exclusion criteria for the review and how studies were grouped for the syntheses.	Page 4
**Information sources**	6	Specify all databases, registers, websites, organisations, reference lists and other sources searched or consulted to identify studies. Specify the date when each source was last searched or consulted.	Pages 3-4
**Search strategy**	7	Present the full search strategies for all databases, registers and websites, including any filters and limits used.	Page 4
**Selection process**	8	Specify the methods used to decide whether a study met the inclusion criteria of the review, including how many reviewers screened each record and each report retrieved, whether they worked independently, and if applicable, details of automation tools used in the process.	Pages 3-4
**Data collection process**	9	Specify the methods used to collect data from reports, including how many reviewers collected data from each report, whether they worked independently, any processes for obtaining or confirming data from study investigators, and if applicable, details of automation tools used in the process.	Page 4
**Data items**	10a	List and define all outcomes for which data were sought. Specify whether all results that were compatible with each outcome domain in each study were sought (e.g. for all measures, time points, analyses), and if not, the methods used to decide which results to collect.	Pages 3-4

Topic	No.	Item	Location where item is reported
	10b	List and define all other variables for which data were sought (e.g. participant and intervention characteristics, funding sources). Describe any assumptions made about any missing or unclear information.	Pages 3-4
**Study risk of bias assessment**	11	Specify the methods used to assess risk of bias in the included studies, including details of the tool(s) used, how many reviewers assessed each study and whether they worked independently, and if applicable, details of automation tools used in the process.	Page 5
**Effect measures**	12	Specify for each outcome the effect measure(s) (e.g. risk ratio, mean difference) used in the synthesis or presentation of results.	Page 5
**Synthesis methods**	13a	Describe the processes used to decide which studies were eligible for each synthesis (e.g. tabulating the study intervention characteristics and comparing against the planned groups for each synthesis (item 5)).	Pages 3-4
	13b	Describe any methods required to prepare the data for presentation or synthesis, such as handling of missing summary statistics, or data conversions.	Pages 3-5
	13c	Describe any methods used to tabulate or visually display results of individual studies and syntheses.	Page 5
	13d	Describe any methods used to synthesize results and provide a rationale for the choice(s). If meta-analysis was performed, describe the model(s), method(s) to identify the presence and extent of statistical heterogeneity, and software package(s) used.	Pages 3-5
	13e	Describe any methods used to explore possible causes of heterogeneity among study results (e.g. subgroup analysis, meta-regression).	Pages 3-5
	13f	Describe any sensitivity analyses conducted to assess robustness of the synthesized results.	Page 5
**Reporting bias assessment**	14	Describe any methods used to assess risk of bias due to missing results in a synthesis (arising from reporting biases).	Page 5

Endosc Int Open | DOI 10.1055/a-2681-2859 | © The Author(s).

Topic	No.	Item	Location where item is reported
**Certainty assessment**	15	Describe any methods used to assess certainty (or confidence) in the body of evidence for an outcome.	Page 5
**RESULTS**			
**Study selection**	16a	Describe the results of the search and selection process, from the number of records identified in the search to the number of studies included in the review, ideally using a flow diagram.	Page 6
	16b	Cite studies that might appear to meet the inclusion criteria, but which were excluded, and explain why they were excluded.	Pages 7-8 and Figure 2
**Study characteristics**	17	Cite each included study and present its characteristics.	Table 1
**Risk of bias in studies**	18	Present assessments of risk of bias for each included study.	Supplementary Table S4
**Results of individual studies**	19	For all outcomes, present, for each study: (a) summary statistics for each group (where appropriate) and (b) an effect estimate and its precision (e.g. confidence/credible interval), ideally using structured tables or plots.	Table 2
**Results of syntheses**	20a	For each synthesis, briefly summarize the characteristics and risk of bias among contributing studies.	Pages 6-9
	20b	Present results of all statistical syntheses conducted. If meta-analysis was done, present for each the summary estimate and its precision (e.g. confidence/credible interval) and measures of statistical heterogeneity. If comparing groups, describe the direction of the effect.	Pages 6-9
	20c	Present results of all investigations of possible causes of heterogeneity among study results.	Pages 7-8
	20d	Present results of all sensitivity analyses conducted to assess the robustness of the synthesized results.	Pages 7-8 and Supplementary Table S3
**Reporting biases**	21	Present assessments of risk of bias due to missing results (arising from reporting biases) for each synthesis assessed.	Page 9 and Supplementary Table S4
**Certainty of evidence**	22	Present assessments of certainty (or confidence) in the body of evidence for each outcome assessed.	Page 9 and Supplementary Table S5
**DISCUSSION**			
**Discussion**	23a	Provide a general interpretation of the results in the context of other evidence.	Pages 9-12

Endosc Int Open | DOI 10.1055/a-2681-2859 | © The Author(s).

Topic	No.	Item	Location where item is reported
	23b	Discuss any limitations of the evidence included in the review.	Pages 11-12
	23c	Discuss any limitations of the review processes used.	Pages 11-12
	23d	Discuss implications of the results for practice, policy, and future research.	Page 12
**OTHER INFORMATION**			
**Registration and protocol**	24a	Provide registration information for the review, including register name and registration number, or state that the review was not registered.	Page 3
	24b	Indicate where the review protocol can be accessed, or state that a protocol was not prepared.	Page 3
	24c	Describe and explain any amendments to information provided at registration or in the protocol.	Page 3
**Support**	25	Describe sources of financial or non-financial support for the review, and the role of the funders or sponsors in the review.	In the submission process
**Competing interests**	26	Declare any competing interests of review authors.	In the submission process
**Availability of data, code and other materials**	27	Report which of the following are publicly available and where they can be found: template data collection forms; data extracted from included studies; data used for all analyses; analytic code; any other materials used in the review.	Fig. 3 and Supplementary Fig. 1-18

Endosc Int Open | DOI 10.1055/a-2681-2859 | © The Author(s).

Supplementary Table 2 Search strategy.

Database	Search strategy
**MEDLINE**	(“intragastric balloon”) AND (“standard medical therapy” OR “standard medical treatment” OR lifestyle OR diet) AND ("randomized controlled trial"[pt] OR "controlled clinical trial"[pt] OR randomized[tiab] OR placebo[tiab] OR "drug therapy"[sh] OR randomly[tiab] OR trial[tiab] OR groups[tiab])
**Embase**	(“intragastric balloon”) AND (“standard medical therapy” OR “standard medical treatment” OR lifestyle OR diet) AND ('randomized controlled trial'/exp OR 'controlled clinical trial'/exp OR randomized:ti,ab OR placebo:ti,ab OR 'drug therapy':lnk OR randomly:ti,ab OR trial:ti,ab OR groups:ti,ab)
**Cochrane**	(“intragastric balloon”) AND (“standard medical therapy” OR “standard medical treatment” OR lifestyle OR diet)

Endosc Int Open | DOI 10.1055/a-2681-2859 | © The Author(s).

Supplementary Table 3 Leave-one-out sensitivity analysis.

Study removed	Pooled effect estimates [95% CI]	Tau²	I², %	*P*
**Percentage of excess weight loss 6 months**				
None	MD 16.80 [9.22-24.38]	95.08	98	0.0001
Farina 2012	MD 17.69 [8.95-26.42]	107.60	98	0.0001
Fuller 2013	MD 15.09 [7.10-23.08]	95.73	98	0.0001
Kashani 2022	MD 13.23 [9.81-16.64]	11.56	74	0.0001
Mohammed 2019	MD 18.30 [10.71-25.90]	79.46	98	0.0001
Ponce 2015	MD 17.35 [8.98-25.71]	98.90	98	0.0001
Sullivan 2018	MD 17.76 [9.47-26.05]	96.52	98	0.0001
Vicente 2020	MD 16.42 [8.07-24.78]	99.53	98	0.0001
**Percentage of excess weight loss 9 months**				
None	MD 14.36 [7.67-21.04]	14.10	49	0.0001
Fuller 2013	MD 12.22 [9.38-15.06]	NA	NA	0.00001
Mohammed 2019	MD 19.80 [9.59-30.01]	NA	NA	0.0001
**Percentage of total body weight loss 6 months**				
None	MD 5.82 [4.42-7.23]	2.62	81	0.00001
Courcoulas 2017	MD 5.60 [4.09-7.11]	2.52	79	0.00001
Farina 2012	MD 6.11 [4.22-8.00]	4.49	83	0.00001
Fuller 2013	MD 5.55 [4.14-6.97]	2.42	81	0.00001
Gómez 2016	MD 5.62 [4.19-7.06]	2.54	82	0.00001
Hollenbach 2024	MD 5.94 [4.45-7.44]	2.78	83	0.00001
Ponce 2015	MD 6.26 [4.60-7.92]	3.21	82	0.00001
Sullivan 2018	MD 6.30 [4.92-7.67]	1.86	69	0.00001
Vicente 2020	MD 5.38 [4.01-6.75]	2.12	79	0.00001
**Percentage of total body weight loss 9 months**				
None	MD 7.66 [3.35-11.96]	13.44	93	0.0005
Abu Dayyeh 2021	MD 5.63 [4.34-6.93]	0.00	0	0.00001
Courcoulas 2017	MD 8.66 [2.49-14.83]	18.42	93	0.006
Fuller 2013	MD 8.69 [2.80-14.56]	17.33	96	0.004
**Percentage of total body weight loss 12 months**				
None	MD 5.33 [4.16-6.50]	0.48	27%	0.00001
Courcoulas 2017	MD 5.76 [4.49-7.03]	0.28	12%	0.00001
Farina 2012	MD 4.54 [3.23-5.85]	0.00	0%	0.00001
Fuller 2013	MD 5.54 [4.27-6.82]	0.50	29%	0.00001
Gómez 2016	MD 5.15 [3.85-6.46]	0.68	39%	0.00001
Hollenbach 2024	MD 5.41 [4.10-6.72]	0.66	38%	0.00001
**Absolute weight loss 6 months**				
None	MD 6.98 [4.80 -9.16]	7.68	87	0.00001

Endosc Int Open | DOI 10.1055/a-2681-2859 | © The Author(s).

Chan 2021	MD 7.77 [5.41-10.11]	7.82	87	0.00001
Courcoulas 2017	MD 7.16 [4.52-9.79]	10.08	88	0.00001
Fuller 2013	MD 6.75 [4.47-9.01]	7.61	88	0.00001
Kashani 2022	MD 7.16 [4.70-9.61]	8.76	89	0.00001
Konopko 2009	MD 6.24 [4.17-8.30]	6.03	86	0.00001
Mohammed 2019	MD 6.85 [4.47-9.22]	7.96	87	0.00001
Sullivan 2018	MD 7.65 [5.31-9.97]	7.47	82	0.00001
Vicente 2020	MD 6.46 [4.28-8.64]	6.84	87	0.00001
**Absolute weight loss 9 months**				
None	MD 8.65 [4.91-12.39]	13.46	95	0.00001
Abu Dayyeh 2021	MD 7.74 [3.00-12.47]	16.52	95	0.001
Courcoulas 2017	MD 9.82 [6.48-13.17]	7.58	88	0.00001
Fuller 2013	MD 9.66 [5.21-14.10]	14.52	96	0.0001
Mohammed 2019	MD 7.45 [3.33-11.56]	12.20	94	0.0004
**Absolute weight loss 12 months**				
None	MD 4.87 [1.90-7.85]	8.28	90	0.001
Chan 2021	MD 5.83 [2.76-8.89]	6.26	87	0.0002
Courcoulas 2017	MD 4.97 [0.58-9.36]	13.77	93	0.03
Farina 2012	MD 3.58 [2.02-5.16]	0.73	38	0.00001
Fuller 2013	MD 5.09 [1.42-8.75]	9.93	93	0.007
**Body mass index loss 6 months**				
None	MD 2.27 [1.53-3.01]	0.79	89	0.00001
Coffin 2017	MD 2.26 [1.38-3.14]	0.97	85	0.00001
Fuller 2013	MD 2.15 [1.37-2.92]	0.78	90	0.00001
Kashani 2022	MD 2.23 [1.44-3.03]	0.82	90	0.00001
Lee 2012	MD 2.45 [1.67-3.23]	0.79	90	0.00001
Mohammed 2019	MD 2.01 [1.27-2.75]	0.66	88	0.00001
Ponce 2015	MD 2.47 [1.58-3.36]	1.01	90	0.00001
Sullivan 2018	MD 2.50 [1.73-3.27]	0.68	82	0.00001
Vicente 2020	MD 2.14 [1.40-2.88]	0.76	90	0.00001
**Body mass index loss 12 months**				
None	MD 2.27 [0.80-3.74]	1.03	91	0.002
Farina 2012	MD 1.50 [0.79-2.21]	NA	NA	0.0001
Fuller 2013	MD 3.00 [2.50-3.50]	NA	NA	0.00001

Endosc Int Open | DOI 10.1055/a-2681-2859 | © The Author(s).

Supplementary Table 4 Risk of bias assessment.

Study ID	D1	D2	D3	D4	D5	Overall
Abu Dayyeh 2021	🟢	🟢	🟢	🟢	🟢	🟢
Fuller 2013	🟢	🟢	🟢	🟢	🟡	🟡
Mohammed 2014	🟡	🟢	🟢	🟢	🟡	🟡
Couroulas 2017	🟢	🟡	🟢	🟢	🟢	🟢
Farina 2012	🟡	🟢	🟢	🟢	🟡	🟡
Hollenbach 2024	🟢	🟢	🟢	🟢	🟢	🟢
Sullivan 2018	🟢	🟢	🟢	🟢	🟢	🟢
Chan 2021	🟢	🟢	🟢	🟢	🟢	🟢
Coffin 2017	🟢	🟢	🟢	🟢	🟢	🟢
Konopko-Zubrzycka 2009	🟡	🟡	🟢	🟢	🟡	🟡
Kashani 2022	🟡	🟢	🟢	🟢	🟡	🟡
Gómez 2016	🟡	🟢	🟢	🟢	🟢	🟢
Lee 2012	🟢	🟡	🟢	🟢	🟡	🟡
Ponce 2015	🟢	🟡	🟢	🟢	🟢	🟢
Vicente 2020	🟢	🟢	🟢	🟢	🟢	🟢

🟢 Low risk
🟡 Some concerns
🔴 High risk

D1 Randomization process
D2 Deviations from the intended Interventions
D3 Missing outcome data
D4 Measurement of the outcome
D5 Selection of the reported result

Endosc Int Open | DOI 10.1055/a-2681-2859 | © The Author(s).

Supplementary Table 5 Certainty of evidence assessment.

Intragastric balloon compared to standard medical treatment for obesity
Population: Patients with obesity.
Intervention: Intragastric balloon.
Control: Standard medical treatment.

Outcomes	No. of patients (studies)	Certainty of the evidence (GRADE)	Anticipated absolute effects: Risk with SMT	Anticipated absolute effects: Risk difference with IGB
% Excess Weight Loss 6 months	1099 (7 RCTs)	⊕⊕⊕◯ Moderate [a]	The mean % Excess Weight Loss was **0** MD	Mean 16.8 MD more (9.22 more to 24.38 more)
% Excess Weight Loss 9 months	202 (2 RCTs)	⊕⊕◯◯ Low [a,b]	The mean % Excess Weight Loss was **0** MD	Mean 14.36 MD more (7.67 more to 21.04 more)
% Excess Weight Loss 12 months	124 (2 RCTs)	⊕⊕⊕◯ Moderate [b]	The mean % Excess Weight Loss was **0** MD	Mean 13.1 MD more (10.43 more to 15.77 more)
% Total Body Weight Loss 6 months	1208 (8 RCTs)	⊕⊕⊕◯ Moderate [a]	The mean % Total Body Weight Loss was **0** MD	Mean 5.82 MD more (4.42 more to 7.23 more)
% Total Body Weight Loss 9 months	617 (3 RCTs)	⊕⊕⊕◯ Moderate [a]	The mean % Total Body Weight Loss was **0** MD	Mean 7.66 MD more (3.35 more to 11.96 more)
% Total Body Weight Loss 12 months	430 (5 RCTs)	⊕⊕⊕⊕ High	The mean % Total Body Weight Loss was **0** MD	Mean 5.33 MD more (4.16 more to 6.5 more)
Absolute Weight Loss 6 months	1126 (8 RCTs)	⊕⊕⊕◯ Moderate [a]	The mean Absolute Weight Loss was **0** MD	Mean 6.98 MD more (4.81 more to 9.15 more)
Absolute Weight Loss 9 months	745 (4 RCTs)	⊕⊕⊕◯ Moderate [a]	The mean Absolute Weight Loss was **0** MD	Mean 8.65 MD more (4.91 more to 12.39 more)
Absolute Weight Loss 12 months	478 (4 RCTs)	⊕⊕⊕◯ Moderate [a]	The mean Absolute Weight Loss was **0** MD	Mean 4.88 MD more (1.9 more to 7.85 more)

Endosc Int Open | DOI 10.1055/a-2681-2859 | © The Author(s).

Body Mass Index Loss 6 months	1182 (8 RCTs)	⨁⨁⨁◯ Moderate [a]	The mean Body Mass Index Loss was **0** MD	Mean 2.27 MD more (1.53 more to 3.01 more)
Body Mass Index Loss 12 months	124 (2 RCTs)	⨁⨁◯◯ Low [a,b]	The mean Body Mass Index Loss was **0** MD	Mean 2.27 MD more (0.8 more to 3.74 more)

*The risk in the intervention group (and its 95% confidence interval) is based on the assumed risk of the comparator group and the relative effect of the intervention (and its 95% CI). **CI:** Confidence interval

GRADE Working Group grades of evidence
High certainty: we are very confident that the true effect lies close to that of the estimate of the effect.
Moderate certainty: we are moderately confident in the effect estimate: the true effect is likely to be close to the estimate of the effect, but there is a possibility that it is substantially different.
Low certainty: our confidence in the effect estimate is limited: the true effect may be substantially different from the estimate of the effect.
Very low certainty: we have very little confidence in the effect estimate: the true effect is likely to be substantially different from the estimate of effect.

Explanations
a. Inconsistency: high heterogeneity.
b. Imprecision: small number of patients.

Endosc Int Open | DOI 10.1055/a-2681-2859 | © The Author(s).

Supplementary Figure S1. Forest plots of percentage of total body weight loss at (A) 6 months, (B) 9 months and (C) 12 months. The intragastric balloon group had a significantly higher percentage of total body weight loss compared to the standard medical treatment group. CI, confidence interval; IGB, intragastric balloon; IV, inverse variance; SD, standard deviation; SMT, standard medical treatment.

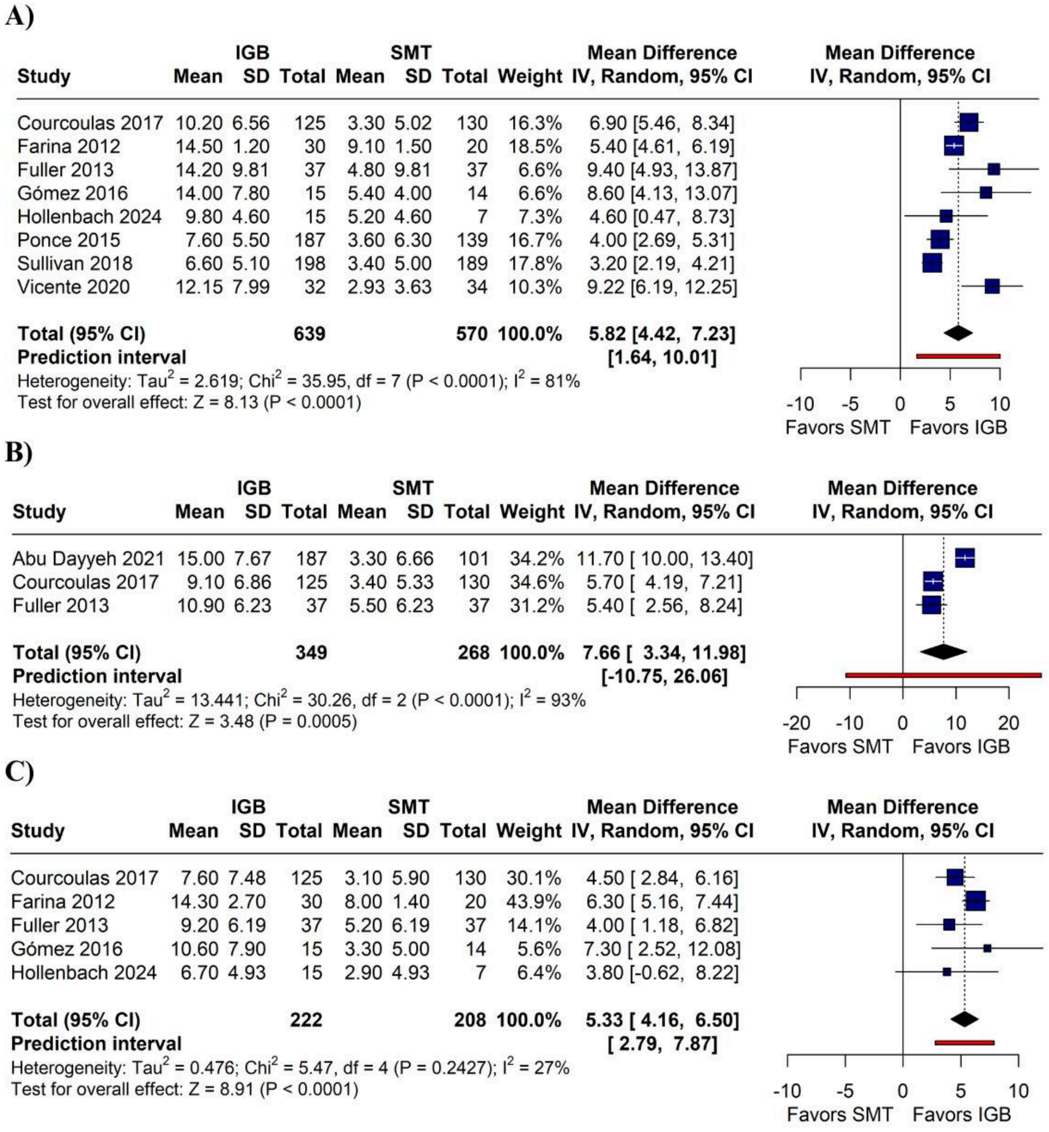

Endosc Int Open | DOI 10.1055/a-2681-2859 | © The Author(s).

Supplementary Figure S2. Forest plots of absolute weight loss at (A) 6 months, (B) 9 months, and (C) 12 months. The intragastric balloon group had significantly higher absolute weight loss compared to the standard medical treatment group. CI, confidence interval; IGB, intragastric balloon; IV, inverse variance; SD, standard deviation; SMT, standard medical treatment.

A)

Study	IGB Mean	IGB SD	IGB Total	SMT Mean	SMT SD	SMT Total	Weight	Mean Difference IV, Random, 95% CI
Chan 2021	9.75	5.12	50	7.48	5.12	49	14.1%	2.27 [0.25, 4.29]
Courcoulas 2017	9.88	6.60	125	3.17	4.82	130	15.1%	6.71 [5.29, 8.13]
Fuller 2013	14.40	10.36	37	5.10	10.36	37	9.2%	9.30 [4.58, 14.02]
Kashani 2022	12.70	5.93	34	6.47	4.63	38	13.3%	6.23 [3.75, 8.71]
Konopko 2009	17.10	8.00	21	3.50	6.40	15	9.2%	13.60 [8.89, 18.31]
Mohammed 2014	14.29	6.44	44	6.39	4.28	84	14.0%	7.90 [5.79, 10.01]
Sullivan 2018	6.60	5.30	198	3.30	5.10	198	15.5%	3.30 [2.28, 4.32]
Vicente 2020	16.20	9.84	32	4.70	8.70	34	9.6%	11.50 [7.01, 15.99]
**Total (95% CI)**			**541**			**585**	**100.0%**	**6.98 [4.80, 9.16]**
**Prediction interval**								**[-0.08, 14.04]**

Heterogeneity: $Tau^2 = 7.677$; $Chi^2 = 55.22$, df = 7 (P < 0.0001); $I^2 = 87\%$
Test for overall effect: Z = 6.28 (P < 0.0001)

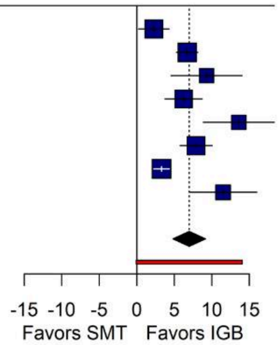

B)

Study	IGB Mean	IGB SD	IGB Total	SMT Mean	SMT SD	SMT Total	Weight	Mean Difference IV, Random, 95% CI
Abu Dayyeh 2021	14.50	7.50	187	3.20	6.50	101	25.5%	11.30 [9.64, 12.96]
Courcoulas 2017	8.79	7.05	125	3.22	0.59	130	26.0%	5.57 [4.33, 6.81]
Fuller 2013	11.00	6.34	37	5.70	6.34	37	23.1%	5.30 [2.41, 8.19]
Mohammed 2014	18.17	4.95	84	5.97	4.39	44	25.4%	12.20 [10.53, 13.87]
**Total (95% CI)**			**433**			**312**	**100.0%**	**8.65 [4.93, 12.37]**
**Prediction interval**								**[-4.49, 21.80]**

Heterogeneity: $Tau^2 = 13.458$; $Chi^2 = 55.82$, df = 3 (P < 0.0001); $I^2 = 95\%$
Test for overall effect: Z = 4.55 (P < 0.0001)

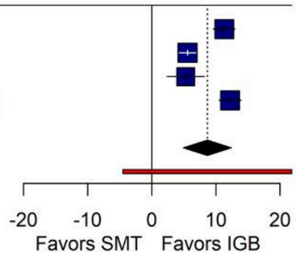

C)

Study	IGB Mean	IGB SD	IGB Total	SMT Mean	SMT SD	SMT Total	Weight	Mean Difference IV, Random, 95% CI
Chan 2021	6.52	5.26	50	4.42	5.26	49	25.0%	2.10 [0.03, 4.17]
Courcoulas 2017	7.34	7.73	125	2.85	5.66	130	26.1%	4.49 [2.82, 6.16]
Farina 2012	17.40	3.40	30	8.90	1.70	20	26.7%	8.50 [7.07, 9.93]
Fuller 2013	9.40	6.46	37	5.30	6.46	37	22.3%	4.10 [1.16, 7.04]
**Total (95% CI)**			**242**			**236**	**100.0%**	**4.87 [1.87, 7.88]**
**Prediction interval**								**[-5.50, 15.25]**

Heterogeneity: $Tau^2 = 8.284$; $Chi^2 = 29.52$, df = 3 (P < 0.0001); $I^2 = 90\%$
Test for overall effect: Z = 3.18 (P = 0.0015)

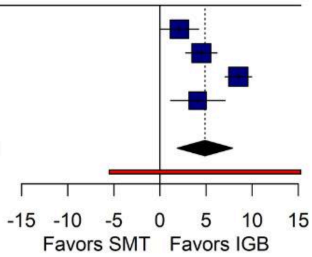

Endosc Int Open | DOI 10.1055/a-2681-2859 | © The Author(s).

Supplementary Figure S3. Forest plots of body mass index loss at (A) 6 months, (B) 12 months. The intragastric balloon group had a significantly higher body mass index loss compared to the standard medical treatment group. CI, confidence interval; IGB, intragastric balloon; IV, inverse variance; SD, standard deviation; SMT, standard medical treatment.

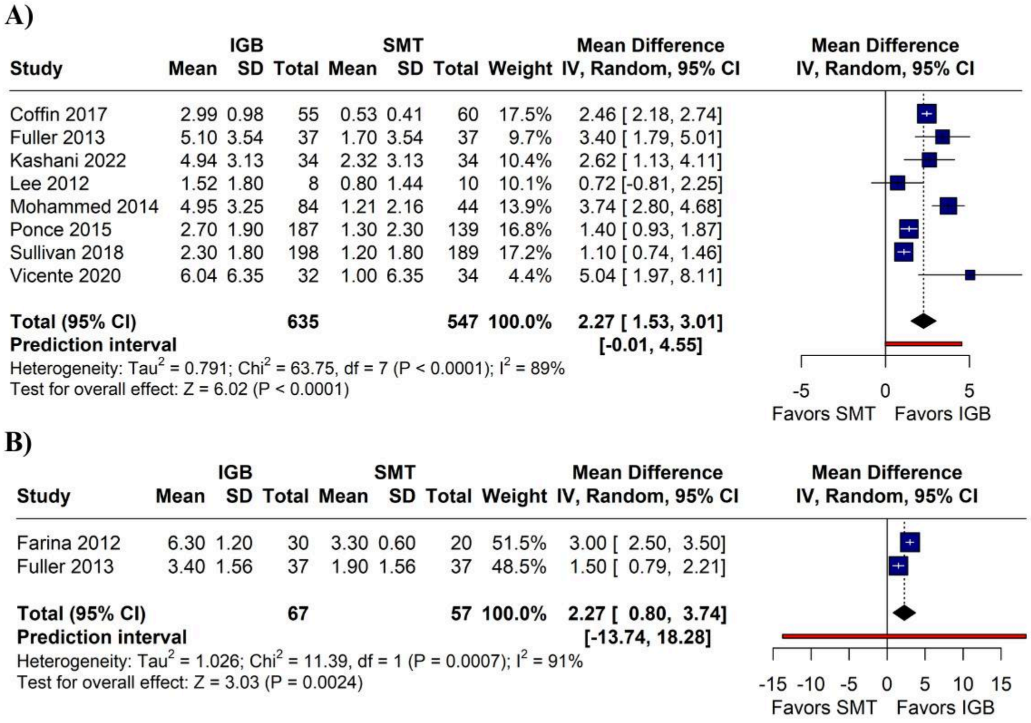

Endosc Int Open | DOI 10.1055/a-2681-2859 | © The Author(s).

Supplementary Figure S4. Subgroup analysis based on the type of balloon for the outcome of percentage of excess weight loss at 6 months. CI, confidence interval; IGB, intragastric balloon; IV, inverse variance; SD, standard deviation; SMT, standard medical treatment.

Study or Subgroup	IGB Mean	IGB SD	IGB Total	SMT Mean	SMT SD	SMT Total	Weight	Mean Difference IV, Random, 95% CI
**Oballoon**								
Sullivan 2018	23.90	19.20	198	12.40	18.80	189	15.0%	11.50 [7.71, 15.29]
Test for overall effect: Z = 5.95 (P < 0.01)								
**Orbera**								
Farina 2012	37.70	3.20	30	25.30	4.10	20	15.6%	12.40 [10.27, 14.53]
Fuller 2013	50.30	34.85	37	16.90	34.85	37	8.1%	33.40 [17.52, 49.28]
Kashani 2022	40.98	1.25	34	15.28	1.19	34	15.8%	25.70 [25.12, 26.28]
Mohammed 2014	14.33	11.37	84	6.40	5.87	44	15.3%	7.93 [4.94, 10.92]
Vicente 2020	27.14	5.75	32	4.54	2.17	34	15.6%	22.60 [20.48, 24.72]
Total (95% CI)			217			169	70.4%	19.07 [11.45, 26.69]
Heterogeneity: $Tau^2 = 67.048$; $Chi^2 = 260.88$, df = 4 (P < 0.01); $I^2 = 98\%$								
**ReShapeDuo**								
Ponce 2015	25.10	21.87	187	11.30	22.40	139	14.5%	13.80 [8.93, 18.67]
**Total (95% CI)**			**602**			**497**	**100.0%**	**17.18 [10.71, 23.65]**
Heterogeneity: $Tau^2 = 68.737$; $Chi^2 = 318.99$, df = 6 (P < 0.01); $I^2 = 98\%$								
Test for overall effect: Z = 5.21 (P < 0.01)								
Test for subgroup differences: $Chi^2 = 3.11$, df = 2 (P = 0.21)								

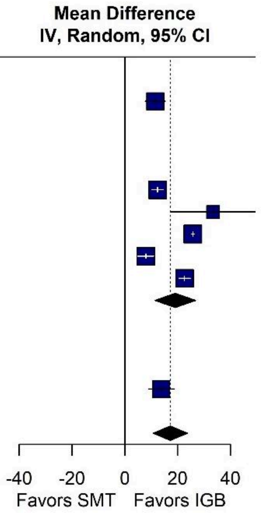

Supplementary Figure S5. Subgroup analysis based on the type of balloon for the outcome of percentage of total body weight loss at 6 months. CI, confidence interval; IGB, intragastric balloon; IV, inverse variance; SD, standard deviation; SMT, standard medical treatment.

Study or Subgroup	IGB Mean	IGB SD	IGB Total	SMT Mean	SMT SD	SMT Total	Weight	Mean Difference IV, Random, 95% CI
**Oballoon**								
Sullivan 2018	6.60	5.10	198	3.40	5.00	189	17.8%	3.20 [2.19, 4.21]
Test for overall effect: Z = 6.23 (P < 0.01)								
**Orbera**								
Courcoulas 2017	10.20	6.56	125	3.30	5.02	130	16.3%	6.90 [5.46, 8.34]
Farina 2012	14.50	1.20	30	9.10	1.50	20	18.5%	5.40 [4.61, 6.19]
Fuller 2013	14.20	9.81	37	4.80	9.81	37	6.6%	9.40 [4.93, 13.87]
Gomez 2016	14.00	7.80	15	5.40	4.00	14	6.6%	8.60 [4.13, 13.07]
Hollenbach 2024	9.80	4.60	15	5.20	4.60	7	7.3%	4.60 [0.47, 8.73]
Vicente 2020	12.15	7.99	32	2.93	3.63	34	10.3%	9.22 [6.19, 12.25]
Total (95% CI)			254			242	65.4%	6.88 [5.40, 8.36]
Heterogeneity: $Tau^2 = 1.587$; $Chi^2 = 12.06$, df = 5 (P = 0.03); $I^2 = 59\%$								
**ReShapeDuo**								
Ponce 2015	7.60	5.50	187	3.60	6.30	139	16.7%	4.00 [2.69, 5.31]
**Total (95% CI)**			**639**			**570**	**100.0%**	**5.82 [4.42, 7.23]**
Heterogeneity: $Tau^2 = 2.619$; $Chi^2 = 35.95$, df = 7 (P < 0.01); $I^2 = 81\%$								
Test for overall effect: Z = 8.13 (P < 0.01)								
Test for subgroup differences: $Chi^2 = 16.45$, df = 2 (P < 0.01)								

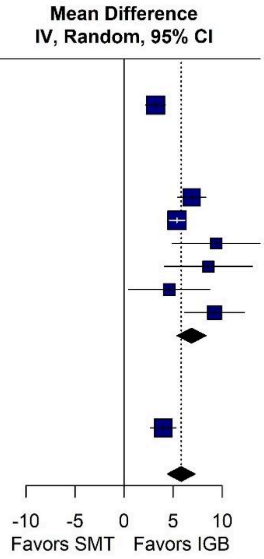

Endosc Int Open | DOI 10.1055/a-2681-2859 | © The Author(s).

Supplementary Figure S6. Subgroup analysis based on the type of balloon for the outcome of percentage of total body weight loss at 9 months. CI, confidence interval; IGB, intragastric balloon; IV, inverse variance; SD, standard deviation; SMT, standard medical treatment.

Study or Subgroup	IGB Mean	IGB SD	IGB Total	SMT Mean	SMT SD	SMT Total	Weight	Mean Difference IV, Random, 95% CI
**Orbera**								
Courcoulas 2017	9.10	6.86	125	3.40	5.33	130	34.6%	5.70 [4.19, 7.21]
Fuller 2013	10.90	6.23	37	5.50	6.23	37	31.2%	5.40 [2.56, 8.24]
Total (95% CI)			162			167	65.8%	5.63 [4.30, 6.97]
Heterogeneity: $Tau^2 = 0$; $Chi^2 = 0.03$, df = 1 (P = 0.85); $I^2 = 0\%$								
Test for overall effect: Z = 8.27 (P < 0.01)								
**Spatz 3**								
Abu Dayyeh 2021	15.00	7.67	187	3.30	6.66	101	34.2%	11.70 [10.00, 13.40]
**Total (95% CI)**			**349**			**268**	**100.0%**	**7.66 [3.34, 11.98]**
Heterogeneity: $Tau^2 = 13.441$; $Chi^2 = 30.26$, df = 2 (P < 0.01); $I^2 = 93\%$								
Test for overall effect: Z = 3.48 (P < 0.01)								
Test for subgroup differences: $Chi^2 = 30.23$, df = 1 (P < 0.01)								

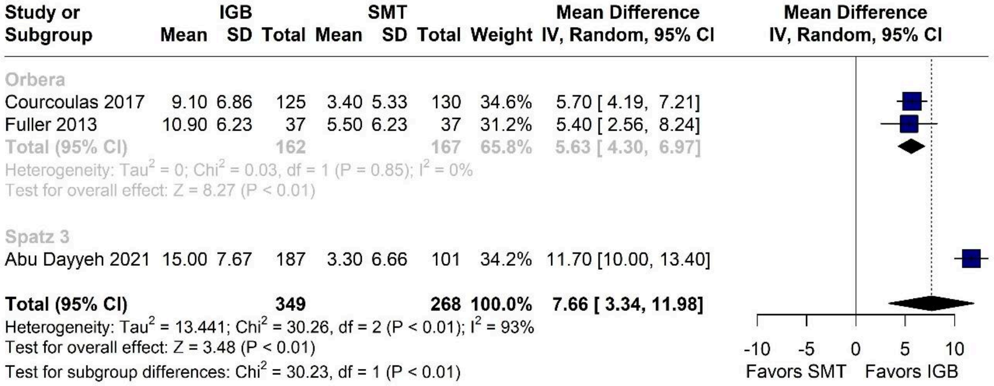

Supplementary Figure S7. Subgroup analysis based on the type of balloon for the outcome of absolute weight loss at 6 months. CI, confidence interval; IGB, intragastric balloon; IV, inverse variance; SD, standard deviation; SMT, standard medical treatment.

Study or Subgroup	IGB Mean	IGB SD	IGB Total	SMT Mean	SMT SD	SMT Total	Weight	Mean Difference IV, Random, 95% CI
**Oballoon**								
Sullivan 2018	6.60	5.30	198	3.30	5.10	189	15.5%	3.30 [2.26, 4.34]
Test for overall effect: Z = 6.24 (P < 0.01)								
**Orbera**								
Chan 2021	9.75	5.12	50	7.48	5.12	49	14.1%	2.27 [0.25, 4.29]
Courcoulas 2017	9.88	6.60	125	3.17	4.82	130	15.0%	6.71 [5.29, 8.13]
Fuller 2013	14.40	10.36	37	5.10	10.36	37	9.1%	9.30 [4.58, 14.02]
Kashani 2022	12.70	5.93	34	6.47	4.63	34	13.2%	6.23 [3.70, 8.76]
Konopko 2009	17.10	8.00	21	3.50	6.40	15	9.2%	13.60 [8.89, 18.31]
Mohammed 2014	14.29	6.44	84	6.39	4.28	44	14.4%	7.90 [6.03, 9.77]
Vicente 2020	16.20	9.84	32	4.70	8.70	34	9.5%	11.50 [7.01, 15.99]
Total (95% CI)			383			343	84.5%	7.64 [5.33, 9.95]
Heterogeneity: $Tau^2 = 7.238$; $Chi^2 = 33.97$, df = 6 (P < 0.01); $I^2 = 82\%$								
**Total (95% CI)**			**581**			**532**	**100.0%**	**6.99 [4.81, 9.16]**
Heterogeneity: $Tau^2 = 7.682$; $Chi^2 = 56.65$, df = 7 (P < 0.01); $I^2 = 88\%$								
Test for overall effect: Z = 6.29 (P < 0.01)								
Test for subgroup differences: $Chi^2 = 11.27$, df = 1 (P < 0.01)								

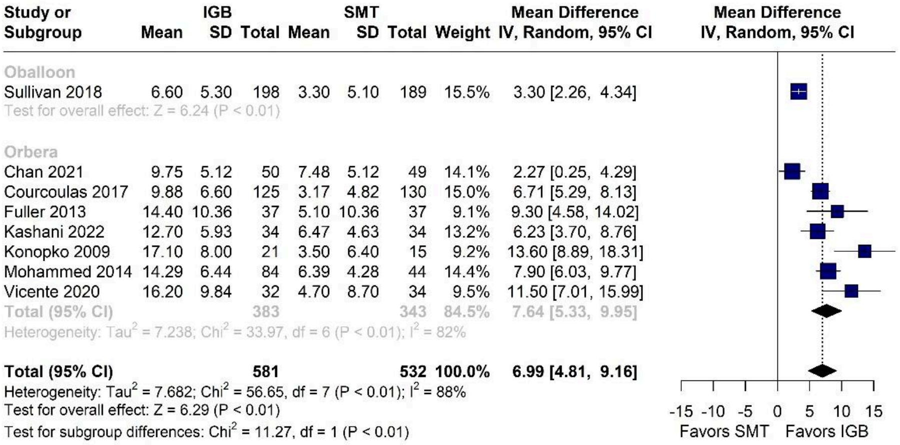

Endosc Int Open | DOI 10.1055/a-2681-2859 | © The Author(s).

Supplementary Figure S8. Subgroup analysis based on the type of balloon for the outcome of absolute weight loss at 9 months. CI, confidence interval; IGB, intragastric balloon; IV, inverse variance; SD, standard deviation; SMT, standard medical treatment.

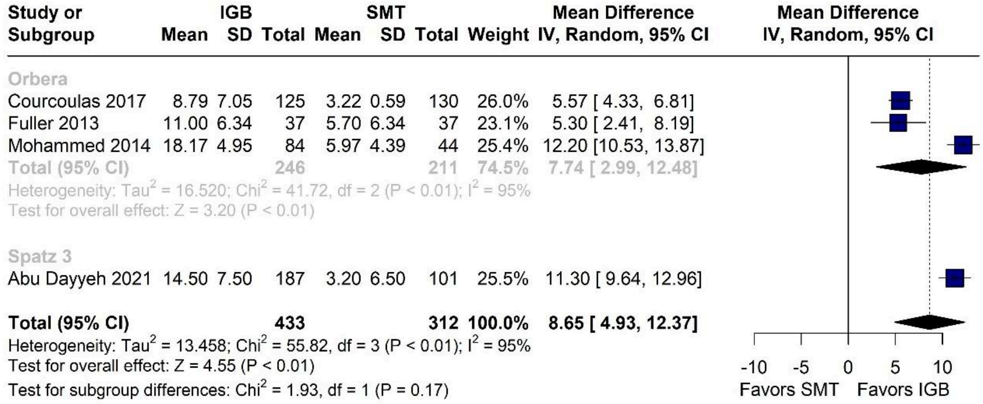

Supplementary Figure S9. Subgroup analysis based on the mean baseline body mass index (≤ 40 kg/m^2 and >40 kg/m^2) for the outcome of percentage of excess weight loss at 6 months. BMI, body mass index; CI, confidence interval; IGB, intragastric balloon; IV, inverse variance; SD, standard deviation; SMT, standard medical treatment.

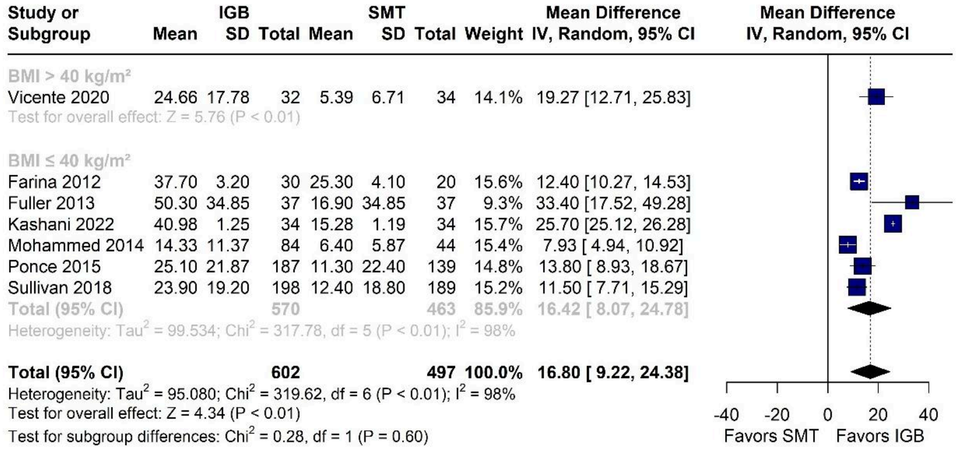

Endosc Int Open | DOI 10.1055/a-2681-2859 | © The Author(s).

Supplementary Figure S10. Subgroup analysis based on the mean baseline body mass index (≤40 kg/m^2 and >40 kg/m^2) for the outcome of percentage of total body weight loss at 6 months. BMI, body mass index; CI, confidence interval; IGB, intragastric balloon; IV, inverse variance; SD, standard deviation; SMT, standard medical treatment.

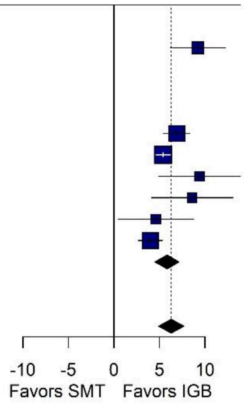

Study or Subgroup	IGB Mean	IGB SD	IGB Total	SMT Mean	SMT SD	SMT Total	Weight	Mean Difference IV, Random, 95% CI
**BMI > 40 kg/m²**								
Vicente 2020	12.15	7.99	32	2.93	3.63	34	11.7%	9.22 [6.19, 12.25]
Test for overall effect: Z = 5.97 (P < 0.01)								
**BMI ≤ 40 kg/m²**								
Courcoulas 2017	10.20	6.56	125	3.30	5.02	130	20.6%	6.90 [5.46, 8.34]
Farina 2012	14.50	1.20	30	9.10	1.50	20	24.5%	5.40 [4.61, 6.19]
Fuller 2013	14.20	9.81	37	4.80	9.81	37	7.0%	9.40 [4.93, 13.87]
Gómez 2016	14.00	7.80	15	5.40	4.00	14	7.0%	8.60 [4.13, 13.07]
Hollenbach 2024	9.80	4.60	15	5.20	4.60	7	7.9%	4.60 [0.47, 8.73]
Ponce 2015	7.60	5.50	187	3.60	6.30	139	21.4%	4.00 [2.69, 5.31]
Total (95% CI)			409			347	88.3%	5.83 [4.53, 7.14]
Heterogeneity: Tau^2 = 1.316; Chi^2 = 13.68, df = 5 (P = 0.02); I^2 = 63%								
**Total (95% CI)**			**441**			**381**	**100.0%**	**6.30 [4.92, 7.67]**
Heterogeneity: Tau^2 = 1.862; Chi^2 = 19.34, df = 6 (P < 0.01); I^2 = 69%								
Test for overall effect: Z = 8.95 (P < 0.01)								
Test for subgroup differences: Chi^2 = 4.06, df = 1 (P = 0.04)								

Supplementary Figure S11. Subgroup analysis based on the mean baseline body mass index (≤ 40 kg/m^2 and >40 kg/m^2) for the outcome of absolute weight loss at 6 months. BMI, body mass index; CI, confidence interval; IGB, intragastric balloon; IV, inverse variance; SD, standard deviation; SMT, standard medical treatment.

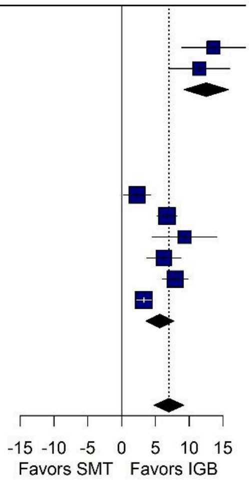

Study or Subgroup	IGB Mean	IGB SD	IGB Total	SMT Mean	SMT SD	SMT Total	Weight	Mean Difference IV, Random, 95% CI
**BMI > 40 kg/m²**								
Konopko 2009	17.10	8.00	21	3.50	6.40	15	9.2%	13.60 [8.89, 18.31]
Vicente 2020	16.20	9.84	32	4.70	8.70	34	9.5%	11.50 [7.01, 15.99]
Total (95% CI)			53			49	18.7%	12.50 [9.25, 15.75]
Heterogeneity: Tau^2 = 0; Chi^2 = 0.4, df = 1 (P = 0.53); I^2 = 0%								
Test for overall effect: Z = 7.54 (P < 0.01)								
**BMI ≤ 40 kg/m²**								
Chan 2021	9.75	5.12	50	7.48	5.12	49	14.1%	2.27 [0.25, 4.29]
Courcoulas 2017	9.88	6.60	125	3.17	4.82	130	15.0%	6.71 [5.29, 8.13]
Fuller 2013	14.40	10.36	37	5.10	10.36	37	9.1%	9.30 [4.58, 14.02]
Kashani 2022	12.70	5.93	34	6.47	4.63	34	13.2%	6.23 [3.70, 8.76]
Mohammed 2014	14.29	6.44	84	6.39	4.28	44	14.4%	7.90 [6.03, 9.77]
Sullivan 2018	6.60	5.30	198	3.30	5.10	189	15.5%	3.30 [2.26, 4.34]
Total (95% CI)			528			483	81.3%	5.65 [3.61, 7.70]
Heterogeneity: Tau^2 = 5.188; Chi^2 = 36.19, df = 5 (P < 0.01); I^2 = 86%								
Test for overall effect: Z = 5.42 (P < 0.01)								
**Total (95% CI)**			**581**			**532**	**100.0%**	**6.99 [4.81, 9.16]**
Heterogeneity: Tau^2 = 7.682; Chi^2 = 56.65, df = 7 (P < 0.01); I^2 = 88%								
Test for overall effect: Z = 6.29 (P < 0.01)								
Test for subgroup differences: Chi^2 = 12.22, df = 1 (P < 0.01)								

Endosc Int Open | DOI 10.1055/a-2681-2859 | © The Author(s).

Supplementary Figure S12. Subgroup analysis based on the mean baseline BMI (≤ 40 kg/m^2 and >40 kg/m^2) for the outcome of body mass index loss at 6 months. BMI, body mass index; CI, confidence interval; IGB, intragastric balloon; IV, inverse variance; SD, standard deviation; SMT, standard medical treatment.

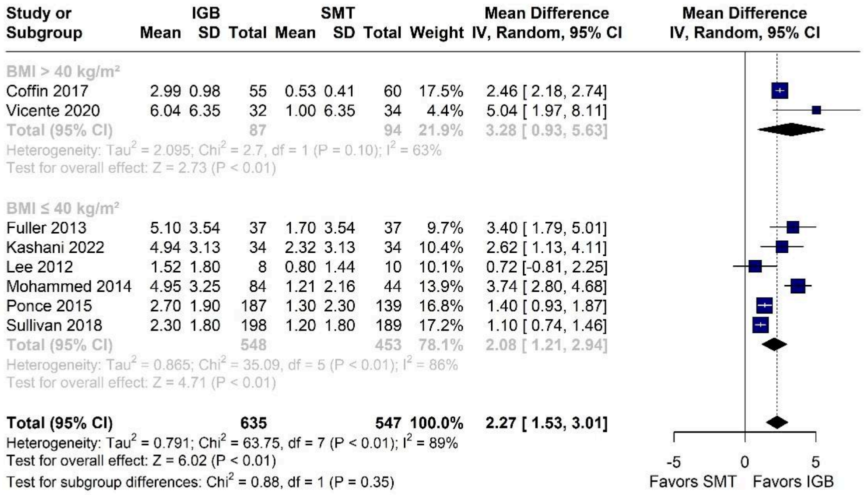

Supplementary Figure S13. Subgroup analysis based on the use of sibutramine in the control group for the outcome of absolute weight loss at 12 months. CI, confidence interval; IGB, intragastric balloon; IV, inverse variance; SD, standard deviation; SMT, standard medical treatment.

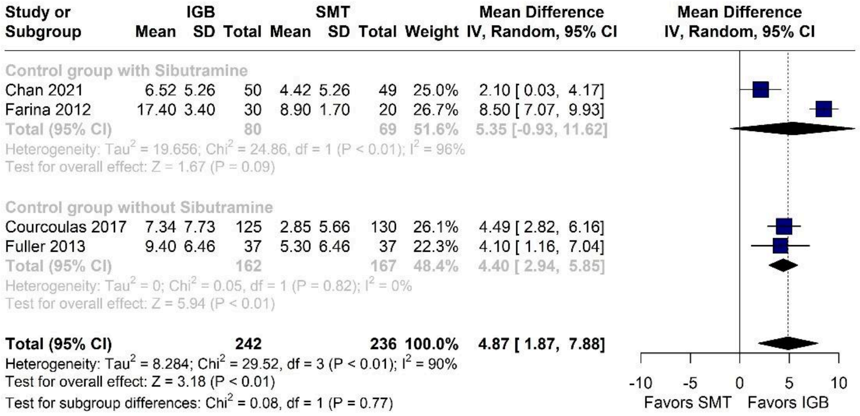

Endosc Int Open | DOI 10.1055/a-2681-2859 | © The Author(s).

Supplementary Figure S14. Meta regression analysis assessing the impact of mean baseline body mass index on 6-month percentage of excess weight loss. BMI, body mass index.

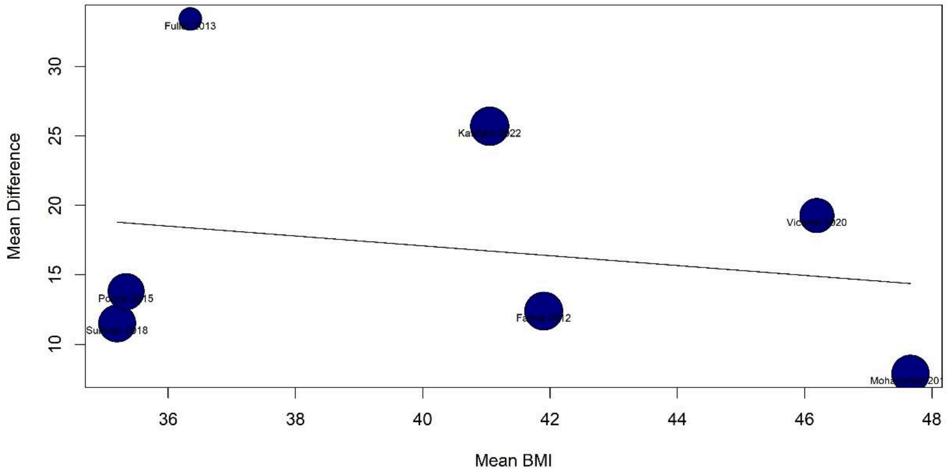

Supplementary Figure S15. Meta regression analysis assessing the impact of mean baseline body mass index on 6-month percentage of total body weight loss. BMI, body mass index.

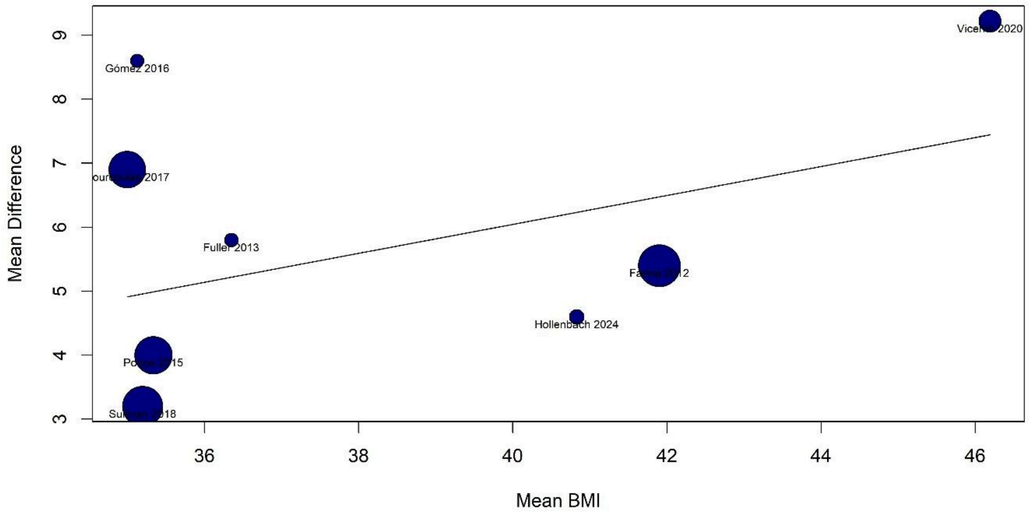

Endosc Int Open | DOI 10.1055/a-2681-2859 | © The Author(s).

Supplementary Figure S16. Meta regression analysis assessing the impact of mean baseline body mass index on 12-month percentage of total body weight loss. BMI, body mass index.

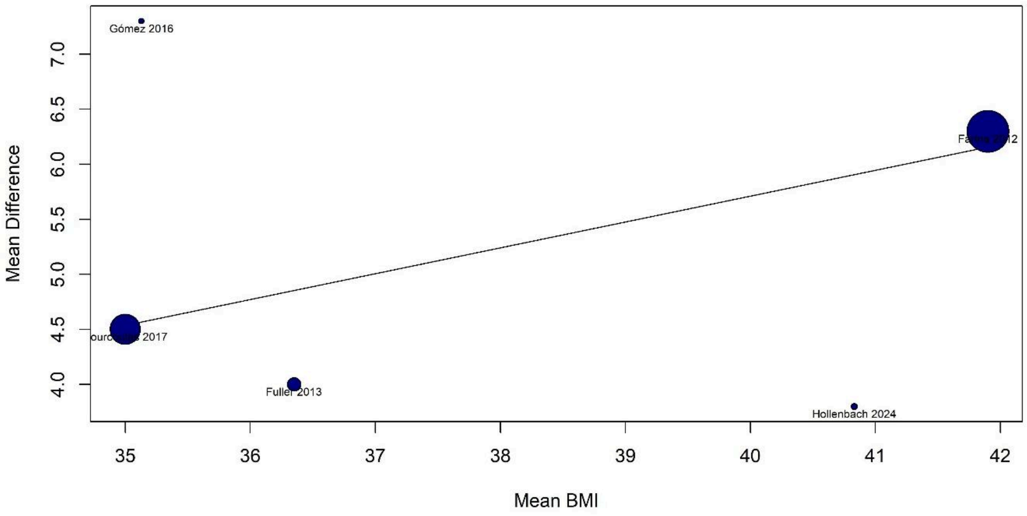

Supplementary Figure S17. Meta regression analysis assessing the impact of mean baseline body mass index on 6-month absolute weight loss. BMI, body mass index.

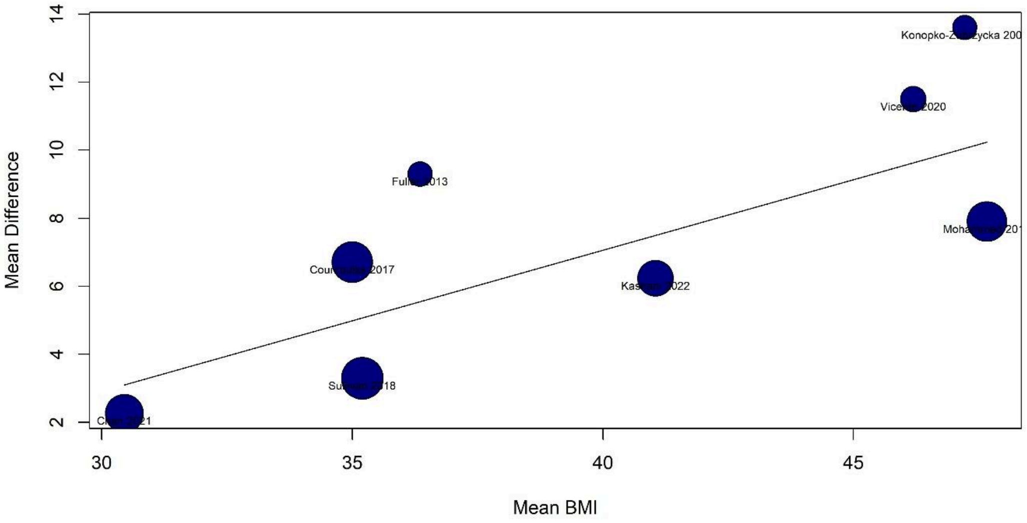

Thieme

Supplementary Figure S18. Meta regression analysis assessing the impact of mean baseline body mass index on 6-month body mass index reduction. BMI, body mass index.

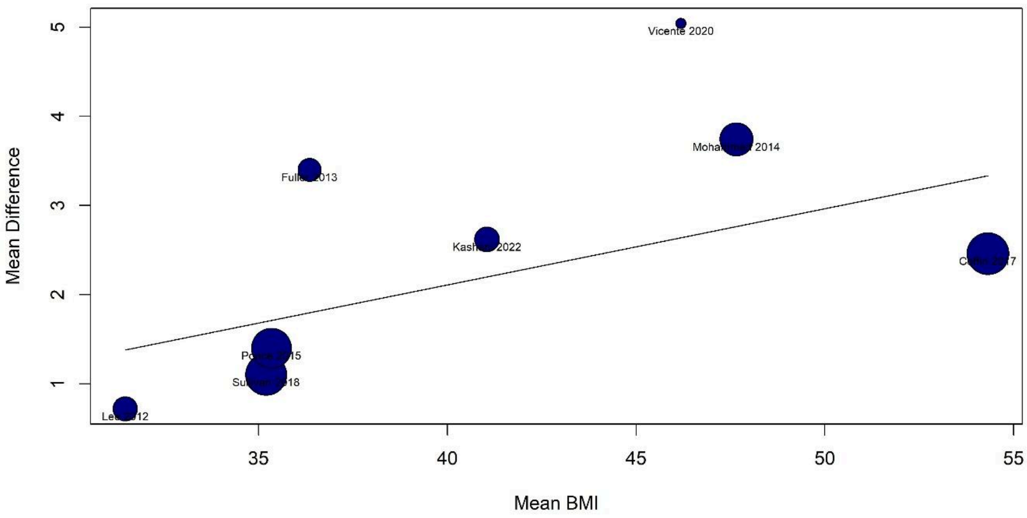

Endosc Int Open | DOI 10.1055/a-2681-2859 | © The Author(s).
